# Human herpesvirus-6, HHV-8 and parvovirus B19 after allogeneic hematopoietic cell transplant: the lesser-known viral complications

**DOI:** 10.1097/QCO.0000000000001020

**Published:** 2024-05-06

**Authors:** Eleftheria Kampouri, Jessica S. Little, Roberto Crocchiolo, Joshua A. Hill

**Affiliations:** a Infectious Diseases Service, Lausanne University Hospital and University of Lausanne, Lausanne, Switzerland; b Dana-Farber Cancer Institute; c Division of Infectious Diseases, Brigham and Women's Hospital, Harvard Medical School, Boston, Massachusetts, USA; d Servizio di Immunoematologia e Medicina Trasfusionale, ASST Grande Ospedale Metropolitano Niguarda, Milan, Italy; e Vaccine and Infectious Disease Division; f Clinical Research Division, Fred Hutchinson Cancer Center; g Department of Medicine, University of Washington, Seattle, Washington, USA

**Keywords:** hematopoietic cell transplant, HHV-6, HHV-8, parvovirus B19, virus

## Abstract

**Purpose of review:**

Viral infections continue to burden allogeneic hematopoietic cell transplant (HCT) recipients. We review the epidemiology, diagnosis, and management of human herpesvirus (HHV)-6, HHV-8 and parvovirus B19 following HCT.

**Recent findings:**

Advances in HCT practices significantly improved outcomes but impact viral epidemiology: post-transplant cyclophosphamide for graft-versus-host disease prevention increases HHV-6 reactivation risk while the impact of letermovir for CMV prophylaxis – and resulting decrease in broad-spectrum antivirals – is more complex. Beyond the well established HHV-6 encephalitis, recent evidence implicates HHV-6 in pneumonitis. Novel less toxic therapeutic approaches (brincidofovir, virus-specific T-cells) may enable preventive strategies in the future. HHV-8 is the causal agent of Kaposi's sarcoma, which is only sporadically reported after HCT, but other manifestations are possible and not well elucidated. Parvovirus B19 can cause severe disease post-HCT, frequently manifesting with anemia, but can also be easily overlooked due to lack of routine screening and ambiguity of manifestations.

**Summary:**

Studies should establish the contemporary epidemiology of HHV-6, and other more insidious viruses, such as HHV-8 and parvovirus B19 following HCT and should encompass novel cellular therapies. Standardized and readily available diagnostic methods are key to elucidate epidemiology and optimize preventive and therapeutic strategies to mitigate the burden of infection.

## INTRODUCTION

Viral infections remain a leading cause of morbidity and non-relapse mortality following allogeneic hematopoietic cell transplant (HCT) [1]. Preventive strategies have substantially reduced the incidence and mitigated the clinical impact of cytomegalovirus (CMV), but such strategies are lacking for other viruses, including human herpesvirus-6 (HHV-6), that continue to burden allogeneic HCT recipients [1]. HHV-6 is ubiquitous, infecting virtually all young children and causing a transient febrile illness (exanthema subitum [sixth disease]). After primary infection, HHV-6 establishes life-long latency and can reactivate in immunocompromised individuals [2]. HHV-6B is responsible for the majority of reactivation and the most frequent cause of infectious encephalitis post-HCT [3,4,5^▪▪^,^6^], while the pathogenic role of HHV-6A is not well established [6,7]. HHV-6 can integrate its genome into telomeres of cell chromosomes as one of its latency mechanisms; when chromosomal integration takes place in germ line cells, vertical transmission can occur to offspring, giving rise to the unique condition of inherited chromosomally integrated HHV-6 (iciHHV-6), in which a copy of the viral genome is present in every nucleated cell [7–10]. The much less ubiquitous HHV-8 (or Kaposi sarcoma-associated herpesvirus, is a potent oncogenic virus implicated in the pathogenesis of several tumors, especially in the setting of advanced HIV infection, which sporadically affects solid organ transplant and allogeneic HCT recipients [11,12]. Parvovirus B19 is another example of a virus commonly causing benign febrile illness with rash (erythema infectiosum [fifth disease]) but with the potential of inducing severe and even chronic disease and reactivation in special patient populations. Due to its distinct tropism for erythroid progenitor cells, red cell aplasia is the most common and feared complication in this setting. Both HHV-8 and parvovirus B19 may be underreported following allogeneic HCT and cause significant morbidity. Herein, we review recent advances in the epidemiology, diagnosis and management of HHV-6B as well as the more obscure HHV-8 and parvovirus B19 in allogeneic HCT recipients. HHV-6A is not reviewed here due to the lack of conclusive evidence associating this virus with reactivation and disease in this setting. 

**Box 1:**
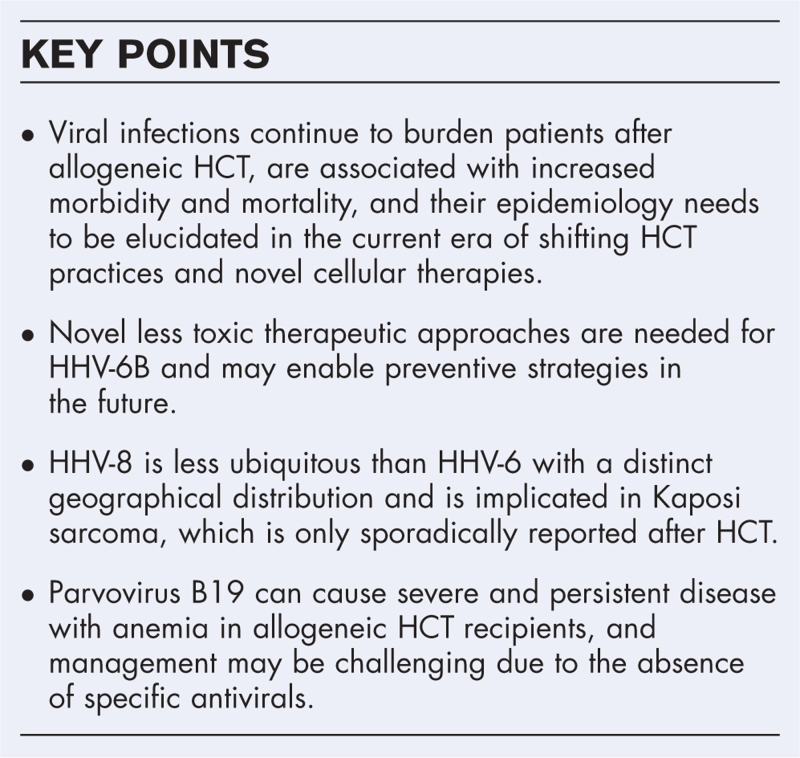
no caption available

## HUMAN HERPES VIRUS-6

### Contemporary epidemiology

Seminal studies report an incidence of HHV-6B reactivation following allogeneic HCT of 40–60% [1,4,13,14], and upwards of 90% in “high-risk” patients receiving umbilical cord blood or T-cell depleted transplants [3,15,16]. These factors along with human leukocyte antigen-mismatched or unrelated donors, acute graft-versus-host disease (GVHD) and treatment with glucocorticoids greatly increase the risk of reactivation and disease [3,13,15,17,18^▪▪^]. HHV-6B encephalitis is the most frequent infectious cause of encephalitis after allogeneic HCT resulting in an attributable mortality of 11% [5^▪▪^], but fortunately remains relatively rare, occurring in 1–3% of HCT recipients and up to 10% following umbilical cord blood transplant [3,15,17]. Seizures and sodium disorders (hyponatremia, syndrome of inappropriate antidiuretic hormone secretion) are more common with HHV-6 encephalitis compared to other viral causes, occurring in 17 and 4% [5^▪▪^]. Importantly, beyond the well defined posttransplant acute limbic encephalitis (PALE) syndrome, HHV-6B can be associated with delirium without encephalitis [19,20], myelitis, and other neurologic manifestations [21–23].

**FIGURE 1:**
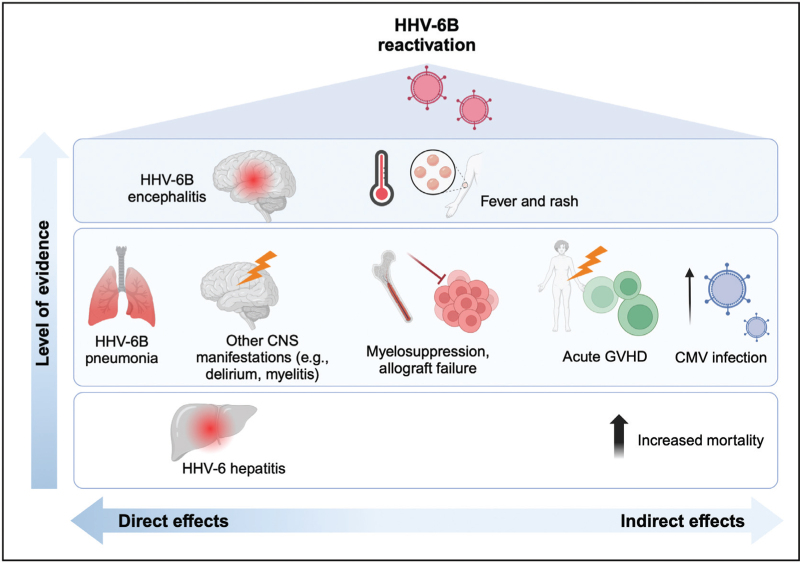
Clinical spectrum of HHV-6B reactivation in allogeneic HCT recipients. The horizontal axis represents direct effects (left) and indirect effects (right) mediated by HHV-6B. The vertical axis represents the level of evidence and strength of association varying from weak (bottom) to strong (top). Figure created with BioRender.com.

The broad cell tropism of HHV-6B *in vivo* leads to diverse clinical manifestations, although causality is not always clear [2]. Epidemiological associations include fever and rash, acute GVHD [3,41], delayed engraftment and allograft dysfunction [12], CMV reactivation [4,24,25], hepatitis [42], and pneumonia (Fig. 1) [21,43,44^▪^]. The pathogenic role of HHV-6B in pneumonia has long been suspected, and recent findings more clearly delineate HHV-6B as a likely cause or contributor to post-HCT pneumonia [26^▪^]. Detection of HHV-6B DNA at a threshold of at least 2.8 log_10_ (≥578) copies/ml in bronchoalveolar fluid was associated with detection of two HHV-6B mRNA transcripts indicative of lytic infection and was an independent risk factor for increased overall mortality and death due to respiratory failure [26^▪^]. Finally, iciHHV-6 occurs in up to 1% of all humans and is characterized by persistently high levels of viral DNA in cellular samples, frequently confounding diagnosis of reactivation and leading to unnecessary interventions [7–10,27]. IciHHV-6 has been linked to increased risk for acute GVHD and CMV reactivation [10,27], and cases of reactivation and disease from the integrated virus have been documented [6,8,28].

Advances in clinical practice and supportive strategies for cellular therapies can impact the epidemiology of HHV-6B. The wider use of posttransplant cyclophosphamide (PTCy) for GVHD prophylaxis has been linked to increased infection risk [18^▪▪^,29^▪^,^30^–^32^]. A study involving 2765 HCT recipients, conducted by the Center for International Blood and Marrow Transplantation Research, found that PTCy use was associated with an increased risk of non-CMV herpesviruses, mainly driven by HHV-6B, increasing non-relapse mortality [29^▪^]. In a recent prospective study involving 208 allogeneic HCT recipients, PTCy use was independently associated with HHV-6 reactivation and clinically relevant HHV-6 infection (defined as the presence of established end-organ disease [6] and other possible manifestations) [18^▪▪^]. Further, the advent of letermovir for CMV prophylaxis after allogeneic HCT was a game-changer for the field and led to a substantial decrease of broad-spectrum antivirals for CMV [33–35]. As these broad-spectrum antivirals have a prophylactic effect against HHV-6B, which is lacking for letermovir, an increase in the incidence or severity of HHV-6B reactivation could be expected [36–39]. A retrospective study among 738 HCT recipients examined HHV-6B reactivation and disease before and after the routine use of letermovir [40^▪^]. While letermovir led to a substantial decrease (40%) in broad-spectrum antiviral use, it did not significantly influence the incidence of HHV-6B reactivation and HHV-6B encephalitis [40^▪^]. Viral kinetics were slightly changed, with HHV-6B detection occurring earlier post-HCT by a median of 10 days and viral load being nearly 1 log_10_ copies/ml higher in plasma and CSF in the postletermovir era [40^▪^]. Whether these changes are clinically relevant remains to be determined in large studies with systematic testing.

### Viral kinetics and why they matter

HHV-6B reactivation occurs early following HCT, with a median time of onset of 3–5 weeks [4,14,18^▪▪^,^36^]. In a prospective study with weekly viral testing among 404 allogeneic HCT recipients, HHV-6B detection was characterized by rapid progression to peak viral loads and quick decline [36]. Sustained HHV-6B detection (≥4 weeks) occurred in approximately one quarter of patients and led to end-organ disease in only 3% [36]. A short time interval from viral detection to end-organ disease of a few days has been reported [36,41,42], but a recent study reported a median time from detection to disease of one month [18^▪▪^]. High plasma viral loads (frequently ≥10 000 copies/ml) are associated with HHV-6B encephalitis, while a dose-dependent link with mortality has also been reported [3,42,43]. Plasma viral loads could be a surrogate for risk-stratification of progression end-organ disease, though certain caveats need consideration. First, there is no universally accepted cut-off to define “high-level” viremia, although plasma viral loads between 1000 and 10 000 copies/ml are most frequently used. A threshold of at least 10 000 copies/ml in plasma appears highly specific for HHV-6 encephalitis in the right clinical context but has low sensitivity [3,42,44^▪^]. Cases of encephalitis have been reported even in the absence of high-level HHV-6 detection (or any detection) in plasma [21,22,38]. Further, the important inter-assay variability – especially prior to the development of a WHO international standard for HHV-6B PCR – hinder generalizability of results and comparability between studies [45,46]. Finally, the short time from detection to progression may limit the utility of serial monitoring as part of preemptive strategies due to the short window of opportunity to preemptively intervene.

### Diagnosis: towards accessibility and standardization of assays

Quantitative assessment of HHV-6B DNA via real-time PCR, ideally discriminating between HHV-6A and HHV-6B, is the recommended diagnostic method [6]. The important inter-assay variability has hindered generalizability of findings of different studies and constitute a major challenge in the field. The development and adoption by labs of the WHO standard for HHV-6 PCR could resolve these hurdles [45,46]. Readily available diagnostic testing for iciHHV-6 is equally important, and its lack in many studies introduces important bias of undiagnosed iciHHV-6 being analyzed with cases of viral reactivation. Droplet digital PCR allows precise quantification of targeted DNA and comparison of copies of viral genome to human cells, and a ratio of nearly 1 copy/cell in cellular samples is considered diagnostic of iciHHV-6; other techniques can also be used [47–49]. Testing of pre-HCT blood samples (or any sample in the donor) can help confirm iciHHV-6 when specific testing is unavailable. Testing for iciHHV-6 is warranted in cases with atypical presentation, high viral loads (often >10^5^–10^6^ copies/ml in cellular samples in the absence of cytopenia), HHV-6A species and/or persistent detection not responding to therapy (>3 weeks or no decrease of viral load after >2 weeks of therapy) [50].

### Treatment and prevention

In the absence of controlled trials, the optimal treatment for HHV-6B is debated, but the role of treatment in reducing HHV-6B encephalitis-related mortality and sequelae is unequivocal [17]. In a study involving 146 allogeneic HCT recipients with HHV-6 encephalitis, the receipt of foscarnet of at least 180 mg/kg/day or ganciclovir of at least 10 mg/kg/day was linked to improved neurological response and lower mortality rates compared to lower doses (56 versus 75%; *P* = 0.022), while no difference between the two agents was observed [17]. Current guidelines recommend treatment with foscarnet 90 mg/kg twice daily (b.i.d.) or ganciclovir 5 mg/kg b.i.d. for at least 3 weeks and until documented clearance from blood, and if possible, clearance from the CSF; combination therapy can be considered in severe cases [6,17,44^▪^,^51^]. Cidofovir may be used as a salvage therapy, but CNS penetration may be suboptimal [6,51].

Preemptive and/or prophylactic strategies are not routinely recommended since the clinical efficacy in preventing progression to end-organ disease has not been demonstrated and current antivirals carry the risk for toxicities tilting the risk-benefit balance against their use [38,41,44^▪^,^52^–^54^]. A retrospective study among 25 umbilical cord blood transplant recipients found prophylactic foscarnet (45 mg/kg twice/day starting on day +7 until engraftment) to be associated with a higher rate of neutrophil engraftment and overall survival at 6 months compared to 61 historical controls, but it did not prevent HHV-6B reactivation [55]. A recent small study evaluated a short course prophylactic foscarnet (60–90 mg/kg/day for 7 days) in 11 allogeneic HCT recipient patients. No end-organ disease occurred, and viremia cleared in all patients, but the lack of a control group and small number precludes conclusions on clinical efficacy [56^▪▪^]. Studies on preventive strategies are summarized in Table 1.

**Table 1:** Studies of preventive strategies using antiviral agents

Study	Design	Participants	Intervention	Details	Outcome	Comment/limitations
Prophylaxis						
El Jurdi *et al*. [55]	Retrospective with historical control	25 UCT (61 controls)	FOS 45 mg/kg 2x/day	From Day +7 until engraftment	Higher rate of neutrophil engraftment and overall 6-month survival; No effect on HHV-6 reactivation	No testing of historical controls
Ogata *et al*. [38]	Prospective multicenter with historical control	57 UCT (63 controls)	FOS 90 mg/kg/day	From Day +7 to +27	Lower rate of high-level HHV-6 reactivation but no effect on HHV-6 encephalitis or acute GVHD	Frequent dose and duration adjustments; insufficient dosing?
Ogata *et al*. [52]	Prospective with historical control	118 UCT or UBMT: 67 with prophylaxis versus 51 controls	FOS 50 mg/kg/day	For 10 days after engraftment	No difference in incidence of high-level HHV-6 reactivation or HHV-6 encephalitis	Breakthrough encephalitis in three patients receiving prophylaxis; insufficient dosing?
Ishiyama *et al*. [54]	Prospective	20: 10 with prophylaxis versus 10 controls	FOS 90 mg/kg/day	From Day +7 to Day +21 (or Day +25 for UCT)	Nonstatistically significant reduction of HHV-6B-related CNS dysfunction	Trend for increased side effects
Tokimasa *et al*. [82]	Retrospective	49 pediatric allo-HCT: 13 with prophylaxis versus 36 controls	GCV 5 mg/kg 2x/day (pre-HCT) 1x/d (post-HCT)	From Day -7 until HCT and from engraftment until D+120	Lower rate of HHV-6 reactivation at 3 weeks post HCT	
Hill *et al*. [39]	Post hoc analysis of multicenter RCT	153 allo-HCT: 92 with prophylaxis versus 61 placebo controls	BCV 100 mg 2x/w orally	Within Day +28 until week 14 post-HCT (min six doses)	Lower hazard for HHV-6 reactivation in adjusted models	BCV unavailable, IV form under development
Preemptive therapy						
Vittayawacharin *et al*. [56^▪▪^]	Prospective, no control group	11 allo-HCT receiving PET	FOS 60–90 mg/kg/day, 7-day	Viral load threshold: ≥1000 copies/ml or >188 copies/ml if cytopenias	Clearance in all patients, recurrence in 1; no encephalitis	Absence of control group and small sample size
Ishiyama *et al*. [53]	Prospective, multicenter	21 UCT and haploidentical HCT: 12 with reactivation, 7 above threshold	FOS 90 mg/kg/day (until resolution on three occasions)	Viral load threshold: ≥500 copies/ml	No difference in outcomes	Potentially more grade 3 adverse events
Ogata *et al*. [41]	Prospective	29 allo-HCT: 19 with reactivation, nine above threshold, six receiving PET	GCV 5–10 mg/kg/day	Viral load threshold: ≥10000 copies/ml	No different in outcomes	Encephalitis developed in two patients before the detection of high-level HHV-6 in plasma

Key studies of prophylactic and preemptive strategies including at least 20 patients are summarized here. Allo-HCT, allogeneic hematopoeitc cell transplant; BCV, brincidofovir; CNS, central nervous system; FOS, foscarnet; GCV, gancilcovir; GVHD, graft versus host disease; PET, preemptive therapy; RCT, randomized controlled trial; UBMT, unrelated bone-marrow transplant; UCT, umbilical cord transplant recipients.

### Expanding the armamentarium

Brincidofovir, a cidofovir prodrug, demonstrates strong in-vitro activity against HHV-6B. In a post hoc analysis of a controlled trial for CMV prophylaxis after HCT, twice-weekly oral brincidofovir significantly reduced HHV-6B reactivation (14 versus 32% in placebo; *P* = 0.019) and lowered viral load among those with reactivation [39].

Adoptive immunotherapy with virus-specific or multivirus-specific T cells products is a promising approach for treatment and prevention of refractory viral infections in immunocompromised patients [57^▪^]. HHV-6B-specific T cells were helpful in treating refractory HHV-6B reactivation after allogeneic HCT in two of three cases [58]. An off-the shelf multivirus-specific T cell product (posoleucel) was found to induce clinical and/or partial virological response in three patients with HHV-6 reactivation [59^▪▪^], and was associated with a lower than expected rate of clinically significant viral infection (14%) when used as prophylaxis in 26 high-risk allogeneic HCT recipients [60^▪▪^]; HHV-6 detection occurred in six patients (23%) without any clinically significant HHV-6 infection [60^▪▪^]. A much anticipated phase 3 randomized controlled prophylaxis trial was unfortunately terminated for futility, although the results and detailed analyses are not yet published [61].

## HUMAN HERPES VIRUS-8

Human herpes virus-8 (HHV-8), also known as Kaposi sarcoma herpesvirus, is a γ-herpesvirus (along with Epstein Barr virus), primarily infecting CD19+ B cells, but also endothelium, CD68+ monocyte macrophage cells, salivary glands, prostate epithelia, and can undergo lytic replication in immunocompromised individuals [11]. Isolated in 1994 from Kaposi sarcoma tissues [62], HHV-8 is an oncogenic virus responsible for neoplastic disease and non-neoplastic manifestations after HCT, including Kaposi's sarcoma, primary effusion lymphoma and Castleman's disease, upon viral reactivation or primary infection [63]. HHV-8 is less ubiquitous than HHV-6 with a seroprevalence varying from upwards of 40% in certain regions (Sub-Saharan Africa) to less than 10% in America and Asia [64]. These geographical differences in HHV-8 seroprevalence mirror the epidemiology of Kaposi sarcoma [65]. Kaposi sarcoma is the most common HHV-8-associated complication after allogeneic HCT, but fortunately occurs only rarely. In a recent study on behalf of the European Group for Blood and Marrow Transplantation (EBMT), Cesaro *et al.*[12] reported an incidence of 0.17 and 0.05% after allogeneic and autologous HCT, respectively. A total of 13 cases of Kaposi sarcoma were reported over a period of 31 years, adding to 17 cases already published as case reports before 2020 [12]. Due to the rarity of this complication, routine serological testing of donor/recipient before HCT or monitoring of HHV-8 viremia after HCT are not recommended [66], but screening of recipients might be considered in high prevalence areas [67]. Among the non-neoplastic complications, a case of HHV-8-associated bone marrow failure has been reported in a patient with non-Hodgkin's lymphoma after autologous HCT [65].

The diagnosis of Kaposi sarcoma or the other HHV-8-associated complications relies on the clinical and histopathological features along with the detection of HHV-8 DNA in tumor tissue or blood as appropriate. Skin lesions are present in most patients, whereas lymph nodes or diffuse (>1 organ or visceral) involvement occur in half of patients diagnosed with Kaposi sarcoma after HCT [12]. Risk factors are not well defined, but Kaposi sarcoma occurred during immunosuppressive treatment for chronic GVHD in almost all reported cases [12].

The treatment of Kaposi sarcoma after allogeneic HCT is based on the withdrawal of immunosuppression whenever possible. Surgical excision of superficial skin lesions or electro-chemotherapy may also be required [63]. Patients with diffuse visceral involvement or unresponsive to immunosuppression withdrawal may benefit from chemotherapy (e.g., anthracyclines) and/or radiotherapy [12,63]. Conversely, the role of antivirals is unclear since Kaposi sarcoma cells harbor latent HHV-8 infection but do not express lytic genes [68]. If immunosuppression cannot be withdrawn, switching to mTOR inhibitor (e.g., sirolimus) may be beneficial because of the antiangiogenetic properties of this class [12,63].

## PARVOVIRUS B19

Human parvovirus B19 is a single-stranded DNA virus that commonly infects children leading to a seroprevalence of more than 60% in adults [69,70]. It is transmitted via respiratory droplets, and rarely via blood transfusion or stem cells, and infects erythroid progenitor cells leading to the widely recognized manifestation of anemia [71,72]. In children, parvovirus B19 presents most commonly as asymptomatic infection or erythema infectiosum, a febrile illness associated with an erythematous malar rash [73,74]. Parvovirus B19 is a significant concern during pregnancy with an increased risk for hydrops fetalis or fetal loss following infection [75]. In non-pregnant immunocompetent adults, infection with parvovirus B19 most often causes arthritis or arthralgias [74], while in immunocompromised hosts including those who have undergone HCT, more serious consequences can occur, including pure red cell aplasia (PRCA), pancytopenia and end-organ disease such as myocarditis, hepatitis or pneumonitis [69,76,77]. HCT recipients can experience both reactivation following a prior primary infection and reinfection during the period of immunosuppression, and prolonged persistence or recurrence has been reported in patients with immune deficits [69,78,79^▪^].

There is limited understanding of the incidence and risk factors for parvovirus B19 after HCT. In one study evaluating adult patients, Eid *et al.*[77] reviewed 98 cases of parvovirus B19 in HCT and SOT recipients. The most common clinical manifestations were anemia (98.8%), leukopenia (37.5%) and fever (25.9%). Rash, arthralgia and organ-invasive disease also occurred in 6–13% of patients [77]. Another study by Schleuning *et al*. [76] found an incidence of 15% among 60 HCT recipients with an associated mortality of 7%. Pediatric studies have described positive parvovirus B19 PCR testing following HCT in up to 7% of recipients, however the majority of these were asymptomatic, while in one study, symptomatic infection was reported in less than 1% [70,80]. Overall, the assessment of parvovirus B19 reactivation and infection after HCT is limited by the lack of routine screening and ambiguity of the presenting symptoms.

Diagnosis of parvovirus B19 is typically made by serology or direct viral detection in blood or tissue via PCR testing, though notably serology may not be reliable in immunocompromised populations with negative IgM results at onset of parvovirus B19 infection in 29% of patients in one study. All but one of those had a positive PCR result suggesting that this may be a more effective test following HCT [69,77]. There are no direct-acting antivirals approved for the treatment of parvovirus B19 infections, and currently, the mainstay of treatment includes intravenous immunoglobulin (IVIG), red blood cell transfusions and reduction of immunosuppression [69,70,81]. Dosing and duration of IVIG varies across studies and institutions and no large studies have established an optimal approach [69,70,81].

## CONCLUSION

The kinetics of HHV-6B reactivation offer key insights into some of the challenges of preemptive approaches but need to be updated in the current era. Novel, less toxic therapeutic modalities may allow preventive strategies. Contemporary epidemiological studies are needed to assess the incidence and impact of major viral infections including HHV-6, and other more insidious pathogens (HHV-8 and Parvovirus B19) following allogeneic HCT and should encompass novel cellular therapies.

## Acknowledgements

*E.K. and J.A.H conceptualized and co-wrote the manuscript, edited the manuscript, and created a figure. J.S.L. and R.C. co-wrote and edited the manuscript.*

### Financial support and sponsorship

*The data that support the findings of this study are available from the corresponding author upon reasonable request.*

### Conflicts of interest

*J.A.H. has served as a consultant for Moderna, Allovir, Gilead, Karius, Geovax, CSL Behring, and received research funding from Allovir, Takeda, Geovax, and Merck. All other authors do not report any conflicts of interest.*

## REFERENCES AND RECOMMENDED READING

Papers of particular interest, published within the annual period of review, have been highlighted as:

▪ of special interest
▪▪ of outstanding interest
